# ATP generation in a host cell in early-phase infection is increased by upregulation of cytochrome c oxidase activity via the p2 peptide from human immunodeficiency virus type 1 Gag

**DOI:** 10.1186/s12977-015-0224-y

**Published:** 2015-11-17

**Authors:** Minako Ogawa, Yuki Takemoto, Shintaro Sumi, Daisuke Inoue, Naoki Kishimoto, Nobutoki Takamune, Shozo Shoji, Shinya Suzu, Shogo Misumi

**Affiliations:** Department of Environmental and Molecular Health Sciences, Faculty of Medical and Pharmaceutical Sciences, Kumamoto University, Kumamoto, 862-0973 Japan; Innovative Collaboration Organization, Kumamoto University, Kumamoto, 860-8555 Japan; Center for AIDS Research, Kumamoto University, Kumamoto, 860-0811 Japan

**Keywords:** p2 peptide, MT-CO, MT-CO1, Intracellular ATP production, HIV-1

## Abstract

**Background:**

Human immunodeficiency virus type 1 (HIV-1) must take advantage of its own proteins with two or more functions to successfully replicate. Although many attempts have been made to determine the function of viral proteins encoded in the HIV-1 genome, the role of the p2 peptide, a spacer between the capsid and the nucleocapsid in HIV-1 Gag in early-phase HIV infection still remains unclarified.

**Results:**

In this study, we show that the p2 peptide enhances HIV-1 acute infection by increasing intracellular ATP production via the activation of mitochondrial cytochrome c oxidase (MT-CO) involved in the respiratory chain. We found that cell-permeable p2-peptide-treated cells were more effectively infected by HIV-1 than control cells. To characterize the effect of the p2 peptide on HIV-1 replication in MAGIC-5 cells, various HIV-1 cDNA products were measured by quantitative real-time PCR. The levels of the late (R/gag), 2-LTR circular (2-LTR), and integrated (Alu) forms of viral cDNAs increased in the presence of the p2 peptide. Interestingly, yeast two-hybrid analysis revealed a novel interaction between the p2 peptide and the mitochondrial intermembrane space domain (N^214^–F^235^) of MT-CO subunit I (MT-CO1). Mutational analysis indicated that Gln^6^ in the p2 peptide is important for the interaction with MT-CO1. The p2 peptide activated MT-CO1 in vitro in a concentration-dependent manner, and fluorescence-microscopy analysis demonstrated that the p2 peptide had a significant effect on mitochondrial targeting. Furthermore, the analysis of HIV-1 lacking a functional p2 peptide demonstrated the inhibition of intracellular ATP production in MT-4 cells and monocyte-derived macrophages (MDMs) and a decrease in reverse transcription efficiency following infection of MT-4 cells and MDMs.

**Conclusions:**

These findings provide evidence that the p2 peptide is a viral positive allosteric modulator of MT-CO and the increased intracellular ATP production after HIV infection in a p2-peptide-dependent manner is essential for efficient reverse transcription in early-phase HIV-1 infection.

## Background

All of the lentiviral Gag proteins contain a spacer peptide between the CA and NC domains [1–5]. In the case of HIV, the spacer peptide p2 is synthesized as part of the Gag and Gag-Pol precursors. Some studies demonstrated that the p2 peptide functions in the late phase of the HIV-1 life cycle. Kaye and Lever reported that the p2 domain of the HIV-1 Gag precursor could contribute to the specific HIV-1 RNA encapsidation [6]. Pettit et al. reported that the p2 domain of the HIV-1 Gag precursor regulates sequential proteolytic processing and ensures the correct assembly of the virion [7]. Our previous study suggests that the p2 peptide is an inherent suicidal inhibitor of HIV-1 protease, because the p2 peptide inhibits the proteolytic cleavage of the recombinant Gag precursor protein into functional structural units [matrix (MA) and CA proteins] in vitro [8]. However, the virological function of the p2 peptide still remains unknown in the early phase of the viral life cycle.

The p2 peptide actually exists in viral particles. Pettit et al. reported that the p2 domain is finally cleaved from the C-terminus of the CA protein to produce fully infectious virions [7]. In our previous study, the p2 peptide was detected in HIV-1 particles by matrix-assisted laser desorption/ionization time-of-flight mass spectrometry [8]. During HIV-1 virion release, each immature virion contains approximately 2400 Gag precursors [9] and approximately 120 Gag-Pol precursors [10], suggesting that a budded mature particle consequently has ∼2400 p2 peptides, which in a 120-nm-diameter sphere corresponds to a p2 peptide concentration of approximately 4 mM. Therefore, the p2 peptide at a high concentration may be focally released into the cytoplasm of target cells after the viral entry.

In this study, we show the virological function of the p2 peptide in the early phase of the viral life cycle. We demonstrated that the p2 peptide released into the cytoplasm during HIV uncoating following the viral entry is targeted towards the mitochondrial intermembrane space domain (N^214^–F^245^) of MT-CO1 involved in the respiratory chain and increases MT-CO activity. Consequently, intracellular ATP content increased in a p2-peptide-dependent manner, which leads to an efficient acute HIV infection. These findings are the first to show that the activity of MT-CO1 is positively regulated by the exogenous viral spacer p2 peptide. These findings reveal a novel regulatory step of early HIV-1 infection.

## Results

### HIV-1 p2 peptide enhanced HIV-1 infection and indirectly enhanced HIV-1 reverse transcription

**Fig. 1:**
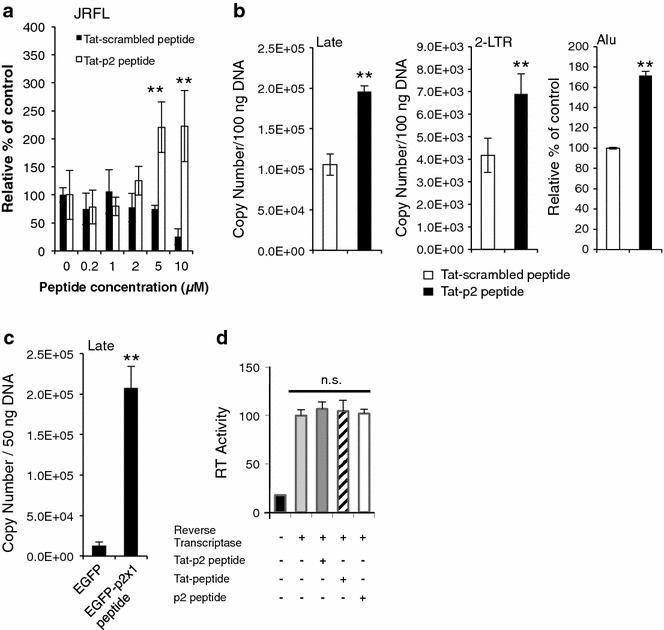
HIV-1 p2 peptide enhanced HIV-1 infection and enhanced HIV-1 reverse transcription. **a** The effect of the p2 peptide on HIV_JRFL_ infection was investigated by MAGIC-5 assay. No significant cytotoxicity of the Tat-p2 peptide- or Tat-scrambled peptide-containing medium was observed. The significance of difference (Student’s *t* test) is indicated as follows: **p < 0.01 vs control. The mean values of at least three independent experiments are shown. **b**, **c** Effect of p2 peptide on reverse transcription in MAGIC-5 cells. Tat-p2-peptide-treated (**b**) or EGFP-p2x1-peptide-transduced (**c**) MAGIC-5 cells were infected with HIV_JRFL_ (**b**, 100 ng of HIV p24) or VSV-G-pseudotyped HIV_NL-CHΔenv WT_ (**c**, 10 ng of HIV p24). The levels of late viral cDNA products (Late), 2-LTR products (2-LTR) or Alu-PCR products (Alu) were determined by quantitative real-time PCR analysis. The significance of difference (Student’s *t* test) is indicated as follows: **p < 0.01. The mean values of at least three independent experiments are shown. The *error bars* denote the standard deviation. **d** Effect of p2 peptide on enzymatic activity of HIV-1 RT. In vitro RT activity assay was performed at a ratio of 1:40 of RT to each peptide (Tat-p2 peptide, Tat-peptide, or p2 peptide). The value in the control experiment (RT only) was set as 100 %. The mean values of at least three independent experiments are shown

During HIV uncoating following the viral entry, the p2 peptide is released from HIV particles into the cytoplasm. To effectively deliver the p2 peptide into cells across the lipid bilayer, a synthetic HIV-1 Tat-p2 peptide (GRKKRRQRRRARVLAEAMSQVTNTATIM) that included residues 359–376 of the HIV-1 Gag precursor fused with the arginine-rich domain (G_48_–R_57_) of HIV-1 Tat was generated [11]. To investigate the effect of the p2 peptide on HIV infection, MAGIC-5 cells [1 × 10^4^ CCR5- and CXCR4-expressing HeLa/CD4(+) cells] were infected with HIV-1_JRFL_ in the presence or absence of the Tat-p2 peptide or Tat-scrambled peptide (GRKKRRQRRRARVLIAVSNMQTTAMATE). The results indicated that the increase in HIV-1_JRFL_ infectivity is dependent on the concentration of the Tat-p2 peptide (Fig. 1a). In contrast, the Tat-scrambled peptide did not increase the HIV-1_JRFL_ infectivity (Fig. 1a).

To clarify the relationship between the increased infectivity and the treatment with the p2 peptide, we carried out quantitative real-time PCR analysis to quantify the late DNA in reverse transcription, 2-LTR circles and integrated proviruses by nested Alu-PCR in Tat-p2-peptide-treated MAGIC-5 cells. The treatment with the Tat-p2 peptide showed significant increases in the levels of the late form (R/gag), 2-LTR circle form and Alu-PCR products (Fig. 1b). These findings suggest that the increased HIV-1 infectivity is linked to a higher efficiency of the late reverse transcription process. Furthermore, we determined whether the levels of the late forms (R/gag) of viral cDNA products are increased by the intracellular expression of the p2 peptide. The p2 peptide was expressed in MAGIC-5 cells as a fusion protein with the p2 peptide fused to the C terminus of EGFP (i.e., the EGFP-p2x1 peptide). As expected, the intracellular expression of the EGFP-p2x1 peptide resulted in a significant increase in the levels of the late forms (R/gag) of viral cDNA products (Fig. 1c), suggesting that the p2 peptide functions as an allosteric effector of HIV reverse transcriptase (RT). However, in vitro RT assay showed that the p2 peptide had no significant direct effect on the enzymatic activity of RT (Fig. 1d). These findings suggest that the p2 peptide is related to some contributory cellular environmental factors that promote reverse transcription.

### HIV-1 p2 peptide interacted with the mitochondrial intermembrane space domain (N^214^–F^235^) of MT-CO1

**Fig. 2:**
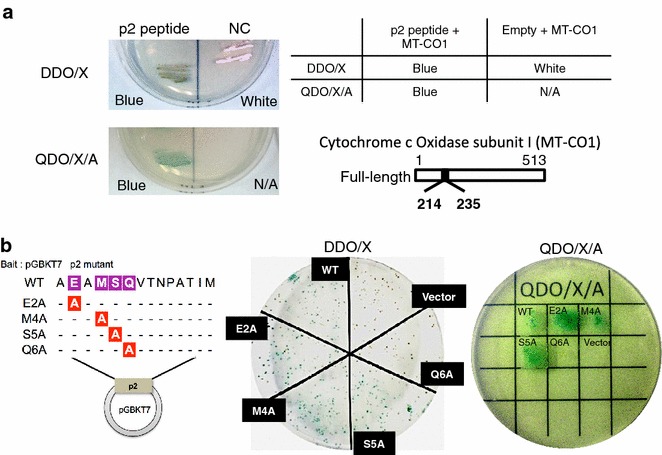
HIV-1 p2 peptide interactd with mitochondrial cytochrome c oxidase. **a** For cotransformation assay using the positive clones from the initial Y2H screening, the bait plasmid (pGBKT7-p2) and prey plasmid encoding the mitochondrial intermembrane space domain (N^214^–F^235^) of MT-CO1 (pGADT7-MT-CO1) were cotransformed into Y2Hgold to confirm the interactions in yeast and grown on DDO/X and QDO/X/A. Growth on QDO/X/A indicated interaction between the p2 peptide and MT-CO1. **b** P2 mutants (E2A, M4A, S5A, and Q6A) were used to investigate their interactions with MT-CO1. The mitochondrial intermembrane space domain (N^214^–F^235^) of MT-CO1 interacted with the mutant E2A, M4A, and S5A p2 peptides, but not with the Q6A mutant p2 peptide

To gain a better understanding of the function of the p2 peptide, we carried out yeast two-hybrid (Y2H) screening to identify cellular factors that potentially interact with the p2 peptide. In the screening, the cDNA sequences of positive clones revealed that the p2 peptide interacted with the mitochondrial intermembrane space domain (N^214^–F^235^) of MT-CO1. To validate the interactions between the p2 peptide and MT-CO1, the bait plasmid (pGBKT7-p2) and the prey plasmid derived from the mitochondrial intermembrane space domain (N^214^–F^235^) of MT-CO1 (pGADT7-MT-CO1) were cotransformed into the yeast strain (Y2HGold), and the yeast was grown on DDO/X (double dropout medium lacking tryptophan and leucine, and supplemented with X-α-Gal) and QDO/X/A (quadruple dropout medium lacking adenine, histidine, tryptophan and leucine, and supplemented with X-α-Gal and aureobasidin A) plates. As shown in Fig. 2a, the growth of blue colonies on both DDO/X and QDO/X/A plates indicated the positive interaction between the p2 peptide and MT-CO1. Furthermore, alanine scanning mutagenesis (E2A, M4A, S5A, Q6A) in the highly conserved AEAMSQ motif of the p2 peptide among HIV-1 group M was carried out to determine the contribution of a specific residue to the interaction between the p2 peptide and MT-CO1. Figure 2b shows that pGBKT7-p2(Q6A) and pGADT7-MT-CO1 cotransformants appeared white on the DDO/X plate and did not grow on the QDO/X/A plate, suggesting that the highly conserved Gln^6^ residue in the AEAMSQ motif is important for the interaction between the p2 peptide and the mitochondrial intermembrane space domain (N^214^–F^235^) of MT-CO1.

### HIV-1 p2 peptide increased MT-CO activity

**Fig. 3:**
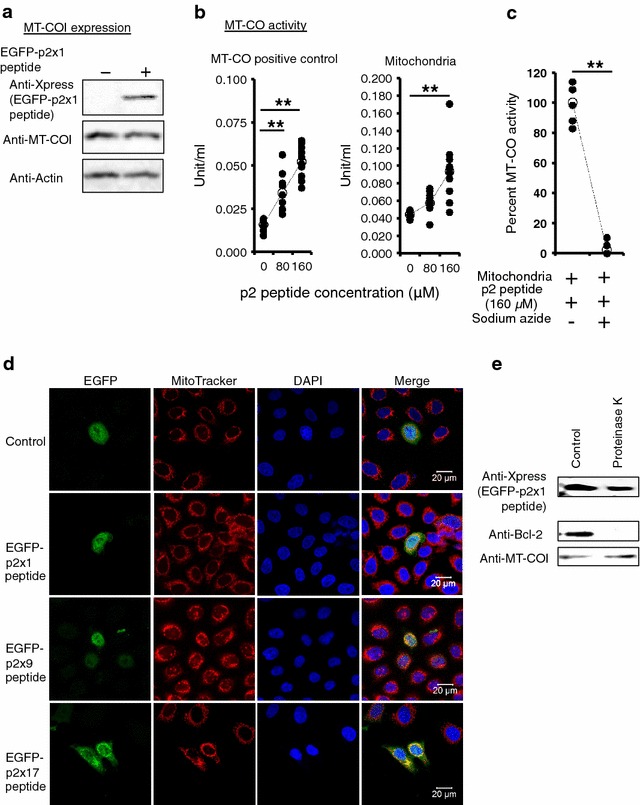
Mitochondrial cytochrome c oxidase was activated by the p2 peptide. **a** MAGIC-5 cells transfected with pcDNA4/EGFP-p2x1 were subjected to western immunoblot analysis for MT-CO1 or actin expression. The expression of the EGFP-p2x1 peptide in MAGIC-5 cells did not affect the expression of both MT-CO1 and actin. A representative result is shown from at least three independent experiments. **b** MT-CO activity measurements show that the p2 peptide activates the enzymatic activity of MT-CO-positive control or native MT-CO1 in mitochondrial fractions from MAGIC-5 cells. Unit Definition: One unit oxidizes one micromole of ferrocytochrome c per minute at 25 °C, pH 7.0. **p < 0.01 versus control; Repeated measures ANOVA and Dunnett’s post hoc test (n = 10). **c** Sodium azide completely inhibited p2-peptide-dependent enzymatic activation of MT-CO. The significance of difference (Student’s *t* test) is indicated as follows: **p < 0.01 (n = 5). **d** Confocal laser scanning microscopy was used for observation of fluorescence in HeLa cells expressing EGFP, or the EGFP-p2x1, EGFP-p2x9, or EGFP-p2x17 peptide. EGFP fluorescence is shown in the left micrographs, MitoTracker-derived fluorescence is shown in the *second column* of the micrographs, and an overlay of EGFP and MitoTracker signals (Merge) is shown in the rightmost column of micrographs. The nuclei were made visible with DAPI staining (*Third column*). EGFP-p2 peptide fusion proteins (EGFP-p2x1 peptide, EGFP-p2x9 peptide, or EGFP-p2x17 peptide) colocalized with the mitochondrial marker MitoTracker. A representative image from three independent experiments is shown. **e** Mitochondria obtained from MAGIC-5 cells expressing the EGFP-p2x1 peptide were incubated with proteinase K (0.05 μg/ml). The lysate of the mitochondrial protein was subjected to SDS-PAGE and examined by western immunoblot analysis using an anti-Xpress, anto-Bcl2 or anti-MT-CO1 antibody. A representative result is shown from at least three independent experiments

To investigate the effect of the p2 peptide on MT-CO1, the expression level of MT-CO1 was analyzed by western immunoblot analysis using an anti-Xpress antibody and MAGIC-5 cells expressing the EGFP-p2x1 peptide. At 48 h post-transfection, no significant change in MT-CO1 expression level was observed (Fig. 3a). Next, to examine the allosteric effect of the p2 peptide on the enzymatic activity of MT-CO1, in vitro cytochrome c oxidase activity assay was performed in accordance with the manufacturer’s instruction manual. The exposures of MT-CO positive control or MT-CO1 in mitochondrial fractions from MAGIC-5 cells to 80 or 160 µM p2 peptide were associated with the positive allosteric effect on MT-CO activity in a concentration-dependent manner (Fig. 3b). In contrast, the p2-peptide-dependent allosteric effect was significantly inhibited by 10 mM sodium azide as a reversible inhibitor of MT-CO (Fig. 3c).

### HIV-1 p2 peptide was targeted to the mitochondrial intermembrane space

To study the mitochondrial localization of the p2 peptide, we examined HeLa cells that were transfected with the pEGFP-p2x1 peptide, pEGFP-p2x9 peptide, or pEGFP-p2x17 peptide, which contained nine or seventeen tandem repeats of the p2 peptide. The expression of the EGFP-p2 peptides was confirmed by confocal microscopy (Fig. 3d). We observed a strong EGFP-p2 peptide-specific signal that partly overlapped with the signal of the mitochondrion-selective marker MitoTracker in a p2-peptide-length-dependent manner (Fig. 3d). To determine whether the EGFP-p2x1 peptide is located inside or outside the mitochondria, we next assessed the sensitivity of the mitochondria-associated EGFP-p2x1 peptide to proteinase K. For this experiment, a freshly prepared mitochondrial fraction was incubated with proteinase K, and the mitochondrial proteins were immunoblotted for the EGFP-p2x1 peptide, Bcl-2, and MT-CO1. As shown in Fig. 3e, MT-CO1, which is embedded in the inner mitochondrial membrane, was fully protected from proteinase K, whereas Bcl-2, which is associated with the external mitochondrial membrane, was completely degraded. Interestingly, a significant fraction of the EGFP-p2x1 peptide was not degraded by proteinase K, suggesting that a portion of the EGFP-p2x1 peptide is located inside the mitochondria and is targeted to the mitochondrial intermembrane space.

### p2-peptide increased intracellular ATP content in MT-4 cells

**Fig. 4:**
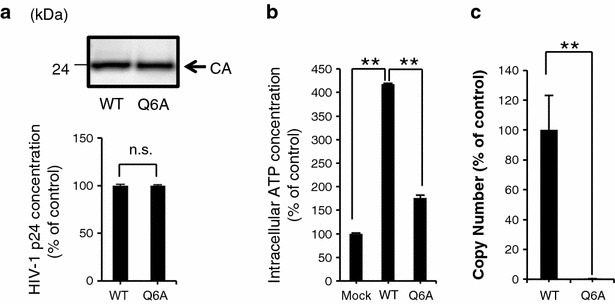
p2-peptide increased intracellular ATP content in MT-4 cells. (**a**, *upper figure*) The p2(Q6A) mutation did not affect normal CA processing. (**a**, *lower figure*) The entry efficiency of the p2(Q6A) mutant virus was determined in terms of the amount of CA protein released into the cytosolic fraction of MT-4 cells (see “Methods”). **b** The infection with the HIV_NL-CH_ _WT_ virus increased the intracellular ATP content in MT-4 cells. In contrast, the intracellular ATP content after HIV_NL-CH p2(Q6A)_ infection was not efficiently increased. **p < 0.01, Nonrepeated measures ANOVA and Dunnett’s test. The mean values of at least three independent experiments are shown.** c** p2(Q6A) mutant virus showed a significant decrease in the level of the late viral cDNA product in MT-4 cells as compared with that in the WT virus. Reverse transcription products were determined by quantitative PCR analysis using late-stage primers (R/gag), as described in “Methods”. To normalize the amount of cellular DNA (100 ng) in the samples, a primer pair complementary to the first exon of the human β-actin gene was used

Because the p2 peptide was associated with the positive allosteric effect on MT-CO activity, we next examined whether HIV-1 infection increases the intracellular ATP content in HIV-1 target cells in a p2-peptide-dependent manner. The CXCR4-tropic p2(Q6A) mutant virus (HIV_NL-CH p2(Q6A)_) showed normal CA processing (Fig. 4a upper figure). To further demonstrate the effect of p2(Q6A) mutation on viral entry, we isolated the cytosolic fraction of target cells postinfection, as described previously by Maréchal et al. [12], who reported that the measurement of cytosolic p24 after virus infection is a reliable assay for the assessment of viral entry events leading to actual cell infection. The viral entry assay indicated that the entry level of p2(Q6A) mutant virus was comparable to that of the WT virus in the cytosolic fraction (Fig. 4a lower figure). However, the p2(Q6A) mutant virus could not efficiently increase the intracellular ATP content in the HIV-1-permissive T cell line MT-4 cells as compared with that in the WT virus at 12 h postinfection (Fig. 4b). Furthermore, the p2(Q6A) mutant virus showed a significant decrease in the level of the late viral cDNA product (Fig. 4c).

### p2-peptide increased intracellular ATP content in MDMs

**Fig. 5:**
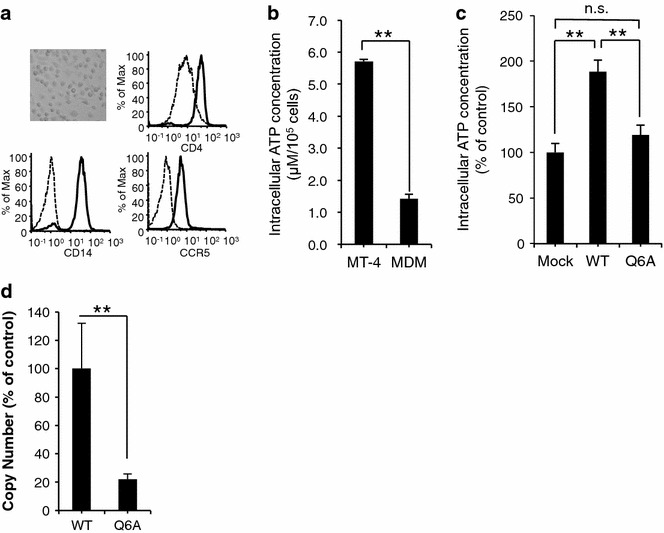
p2-peptide increased intracellular ATP content in MDMs. **a** Cell morphology and expression of surface receptors on MDMs. Freshly prepared MDMs were stained with monoclonal antibodies specific for CD14 (MAb M5E2), CD4 (MAb OKT4), or CCR5 (MAb 45531.111) and analyzed by flow cytometry. **b** Comparison of intracellular ATP level between MT-4 and MDMs. **c** The infection with the CCR5-tropic HIV_AD8_ _WT_ virus increased the intracellular ATP content in MDMs. In contrast, the intracellular ATP content after HIV_NL-CH(AD8) p2(Q6A)_ infection was not significantly increased compared with mock treatment. **p < 0.01, Nonrepeated measures ANOVA and Dunnett’s test. The mean values of at least three independent experiments are shown. **d** p2(Q6A) mutant virus showed a significant decrease in the level of the late viral cDNA product in MDMs as compared with that in the WT virus. MDMs were infected with VSV-G-pseudotyped HIV_NL-CHΔenv p2(Q6A)_. Reverse transcription products were determined by quantitative PCR analysis using late-stage primers (R/gag), as described in “Methods”. To normalize the amount of cellular DNA (100 ng) in the samples, a primer pair complementary to the first exon of the human β-actin gene was used

Next, we studied whether this was also the case in the other major target of HIV-1, primary macrophages. Monocytes were cultured in differentiation medium and the resulting MDMs were characterized on the fifth day of differentiation in terms of the functional marker CD14, CD4, and CCR5 by flow cytometric analysis. As shown in Fig. 5a, the prepared cells showed a typical morphology of MDMs, and CD4 and CCR5 were virtually detected on CD14-positive MDMs. ATP bioluminescence assay demonstrated that the intracellular ATP content in MDMs was about four fold lower than that of MT-4 cells in the absence of HIV infection (Fig. 5b). Furthermore, as expected, the CCR5-tropic p2(Q6A) mutant virus (HIV_AD8 p2(Q6A)_) could not efficiently increase the intracellular ATP content in MDMs as compared with that in the WT virus at 12 h postinfection (Fig. 5c). Finally, the single-round replication-defective VSV-G-pseudotyped HIV_NL-CHΔenv p2(Q6A)_ (10 ng of HIV p24) also showed a significant decrease in the level of the late viral cDNA product (Fig. 5d). These findings show that the p2 peptide plays a key role in the increase in intracellular ATP content in HIV target cells.

## Discussion

Although many attempts have been made to determine the function of viral proteins encoded in the HIV-1 genome, the role of the p2 peptide in early-phase HIV infection has remained unclarified because it has been very difficult to study the peptide owing to a lack of an efficient assay for this viral low-molecular-weight p2 peptide. In this study, the cell-permeable p2-peptide-treated cells were more effectively infected with HIV-1 than control cells (Fig. 1). The p2-peptide-dependent increase in HIV-1 infectivity is associated with a higher efficiency of the late reverse transcription process (Fig. 1b). However, in vitro RT assay showed that the p2 peptide showed no significant direct effect on the enzymatic activity of RT (Fig. 1d). The effect of treatment with a higher concentration of the p2 peptide (50–250 µM) on reverse transcription suggests that the p2 peptide may interact with a host saturable target because the effect was saturated (data not shown). Therefore, we used the Y2H system because it is a very powerful tool for identifying unknown host-binding targets. Y2H analysis revealed the novel interaction between the p2 peptide and MT-CO1, and that Gln^6^ in the p2 peptide was important for the interaction with MT-CO1 (Fig. 2b). To the best of our knowledge, the direct binding of the p2 peptide to MT-CO1 has not been reported yet.

**Fig. 6:**
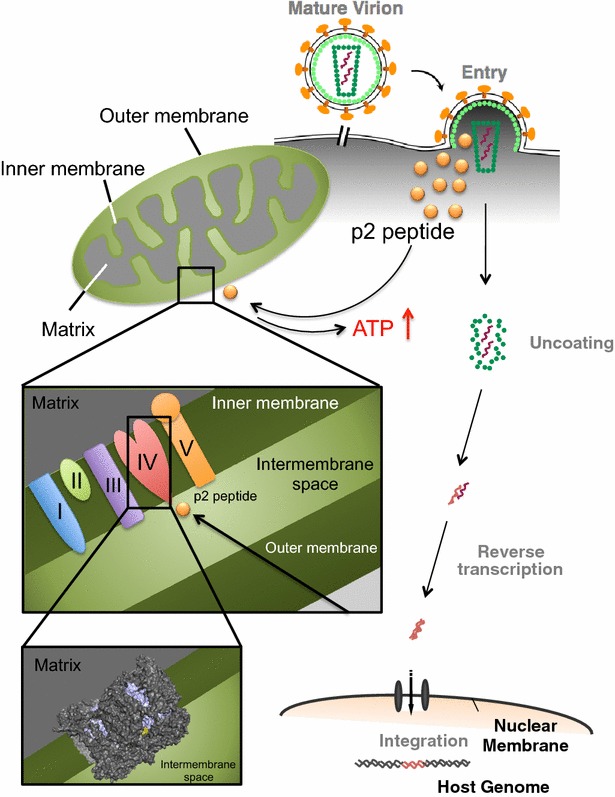
Schematic illustration showing the mechanism by which p2 peptide positively regulates HIV-1 infection. Although the actual molecular basis of the allosteric mechanism of the p2 peptide is still unclear, the findings of this study suggest that the p2 peptide is a viral positive allosteric modulator of MT-CO1 and more efficiently supplies ATP for the active nuclear import of HIV-1 PIC. The N^214^–F^235^ domain (shown in *yellow* in the *lower inset*) was completely conserved between human and bovine MT-CO1, and the crystal structure analysis of bovine heart MT-CO demonstrated that the domain is exposed on the intermembrane space side of the inner membrane. MT-CO-1 was shown in *purple* inside cytochrome c oxidase

MT-CO1 is the main subunit of the MT-CO complex. Although the crystal structure of human MT-CO has not yet been reported, Tsukihara et al. demonstrated that bovine MT-CO is composed of 13 subunits [13]. The three major subunits (MT-CO1, MT-CO2, and MT-CO3) form the catalytic core and are encoded by mitochondrial DNA. The remaining subunits are nucleus-encoded. The N^214^–F^235^ domain was completely conserved between human and bovine MT-CO1, and the crystal structure analysis of bovine heart MT-CO [13] demonstrated that the domain is exposed on the intermembrane space side of the inner membrane (Fig. 6, shown in yellow in the lower inset), suggesting that the p2 peptide is readily accessible to the domain of MT-CO1 (N^214^–F^235^). Actually, the p2 peptide directly activated the MT-CO positive control and native MT-CO in mitochondrial fractions in vitro (Fig. 3b). Furthermore, the WT virus efficiently increased the intracellular ATP content in MT-4 cells and MDMs (Figs. 4b, 5c). These findings are the first demonstration of the functional interaction between the p2 peptide and MT-CO1 and this interaction plays a key role in increasing intracellular ATP production in the target cell.

The nuclear import of the HIV-1 preintegration complex (PIC) is an ATP-dependent process [14, 15], which suggests the involvement of the host cell nuclear transport machinery and viral and host nuclear localization signals. HIV-1 PIC contains at least four karyophilic proteins [viral (MA, IN, and Vpr) and host cell (LEDGF/p75) proteins]. Many studies demonstrated that the passage of HIV-1 PIC through the nuclear envelope is a necessary step in HIV infection of both dividing and nonproliferating cells [16–18]. Bukrinsky et al. demonstrated that the lack of two-LTR circle formation (as a marker of entry of viral DNA into the nucleus) under intracellular low-ATP-content conditions is a consequence of the inhibition of nuclear import of HIV-1 DNA [14]. Furthermore, they reported that the synthesis of HIV-1 DNA in HIV-1-infected cells was less efficient in ATP-depleted cell cultures [14]. Consistent with these findings, the treatment with the Tat-p2 peptide showed significant increases in the levels of 2-LTR circle products, which are generally used as a marker of PIC nuclear import (Fig. 1b). Furthermore, Figs. 4c and 5d show that the inhibition of an effective increase in intracellular ATP content in target cells decreased reverse transcription efficiency during the acute infection of the p2(Q6A) mutant virus. These findings suggest that the p2 peptide more efficiently supply ATP for active nuclear import of HIV-1 PIC as a result of binding of the p2 peptide to MT-CO1 (Fig. 6).

## Conclusions

We conclude that the p2 peptide is a viral positive allosteric modulator of MT-CO involved in the respiratory chain and more efficiently supplies ATP for active nuclear import of the HIV-1 preintegration complex. These are the initial findings showing that the enzymatic activity of MT-CO1 is positively regulated by the exogenous viral spacer p2 peptide. This study reveals a novel regulation step of early HIV-1 infection.

## Methods

### Cell culture

MAGIC-5 cells, which were engineered to express CCR5 in HeLa-CD4-LTR/β-Gal cells by transfection with an expression vector for CCR5, were maintained at 37 °C in DMEM supplemented with 5 % fetal calf serum (FCS) containing 0.2 mg/ml G418 (Sigma-Aldrich Co., LLC), 50 U/ml hygromycine (Sigma-Aldrich Co., LLC), 1 µg/ml blasticidin A (Kaken Pharmaceutical Co., Ltd.), and 1 µg/ml puromycin (Sigma-Aldrich Co., LLC) in 5 % CO_2_. T-cell line MT-4 cells, HEK293 cells, and 293T cells were maintained at 37 °C in RPMI-1640 and DMEM supplemented with 10 % FCS containing 100 IU/ml penicillin and 100 μg/ml streptomycin in 5 % CO_2_. TZM-bl cells were obtained from the NIH AIDS Research and Reference Reagent Program. TZM-bl cells are HeLa cell clones that were engineered to express CD4 and CCR5 and contain integrated reporter genes for firefly luciferase and *E*. *coli* β-galactosidase under the control of HIV-1 LTR, permitting the sensitive and accurate measurements of infection. Human monocyte-derived macrophages (MDMs) were prepared in accordance with the following protocol. PBMCs obtained using PANCOLL reagent (PAN Biotech, Aidenbach, Germany) were suspended in RPMI 1640 medium supplemented with 1 % FCS at a density of 1 × 10^6^ cells/ml, and seeded in dishes. Monocytes were enriched by allowing them to adhere to the dishes for 1 h at 37 °C, and non-adherent cells were removed by extensive wash with PBS. Next, the adherent monocytes were differentiated into macrophages by culturing them with RPMI 1640 supplemented with 10 % FCS containing 100 ng/ml rhM-CSF. After 3 days, the cultures were replaced with fresh complete media after extensive wash with PBS to remove nonadherent cells, and incubated for another 2 days. At day 5, the purity of MDMs was routinely >95 % according to a flow cytometric analysis of CD14 expression. Furthermore, the expression of CD4 and CCR5 was also confirmed by flow cytometry analysis.

### Plasmids

The coding region of HIV-1 p2 was amplified by PCR using the primers P2-UP (5′-AGGATCCGCTGAAGCAATGAGCCAAGTA-3′) and P2-DN1 (5′-TTTAGATCTTTACATTATGGTAGCTGGATTTGT-3′), and cloned into the *Pst*I and *Eco*RV sites of the pcDNA4/HisMaxC plasmid (Life Technologies Corporation) (pcDNA4/HisMaxC-p2). Then, the coding sequence of the EGFP gene was cloned into the *Eco*RI and *Pst*I sites of pcDNA4/HisMaxC-p2 (pcDNA4/EGFP-p2x1). Furthermore, the tandem repeats of DNA fragments coding the p2 region (p2x9 and p2x17) were also amplified by a PCR-based method using the primers P2-UP (5′-AGGATCCGCTGAAGCAATGAGCCAAGTA-3′), P2-DN1 (5′-TTTAGATCTTTACATTATGGTAGCTGGATTTGT-3′), and P2-DN2 (5′-TTT AGA TCT TCC GAT TAT GGT AGC TGG ATT TGT-3′). The amplified DNA fragments were cloned into pcDNA4/HisMaxC with the coding sequence of the EGFP gene. In pcDNA4/EGFP-p2x9 or pcDNA4/EGFP-p2x17, individual p2 peptides were separated by the linker sequence Gly-Arg-Ser.

### Viruses

The clade B laboratory-adapted strain HIV-1_JRFL_ was used. The virus was propagated in a chronically HIV-1_JRFL_-infected T cell line (Molt4-CCR5/JRFL) grown in a complete medium consisting of RPMI 1640 supplemented with 10 % heat-inactivated defined FBS (HyClone), penicillin (100 IU/ml), and streptomycin (0.1 mg/ml). Single-round replication-defective VSV-G-pseudotyped HIV_NL-CHΔenv WT_ was produced by cotransfection of 293T cells with pHCMV-G and pNL-CHΔenv [19, 20]. Furthermore, the infectious molecular clone pNL-CH [20], derived from the pNL4-3 clone of HIV-1, was used to prepare the CXCR4-tropic p2(Q6A) mutant virus. Point mutation was introduced into the p2 domain in pNL-CH by site-directed mutagenesis. The *Spe*I-*Apa*I fragment of the p2(Q6A) mutant was recloned into the full-length HIV expression plasmid pNL-CH_*Spe*I-*Apa*I_, which did not have the backbone *Spe*I-*Apa*I fragment. Finally, HIV_NL-CH p2(Q6A)_ was produced by the transient transfection of HEK293 cells using pNL-CH p2(Q6A). Four days after transfection, the virus-containing supernatant was collected and clarified by filtration through 0.45-μm-pore-size filters. In addition, the CCR5-tropic p2(Q6A) mutant virus (HIV_AD8 p2(Q6A)_) was produced using pNL(AD8) [21] derivative that carries Q6A mutation in the p2 domain. Finally, VSV-G-pseudotyped HIV_NL-CHΔenv p2(Q6A)_ was produced by cotransfection of 293T cells with pHCMV-G and pNL-CHΔenv p2(Q6A).

### MAGIC-5 assay

Viral infectivity was determined using MAGIC-5 cells [22]. The cells were seeded (1 × 10^4^ cells per well) and then infected with the HIV_JRFL_ strain [2.16 × 10^5^ tissue culture infective dose_50_ (TCID_50_)/ml, multiplicity of infection = 0.01] in the presence of the Tat-p2 peptide (GRKKRRQRRRARVLAEAMSQVTNTATIM) [11] or the Tat-scrambled peptide (GRKKRRQRRRARVLIAVSNMQTTAMATE) at the indicated concentrations and DEAE-dextran (20 μg/ml) for 48 h. The cells were fixed with 1 % formaldehyde-0.2 % glutaraldehyde in PBS for 5 min, washed, and then stained with X-gal. Control experiments were carried out under identical conditions in the presence of the Tat-scrambled peptide. The number of cells stained blue was expressed as a percentage (%) relative to the number of cells stained blue in the control.

### Quantitative analysis of HIV-1 reverse transcription during acute infection

De novo synthesized HIV-1 cDNA was analyzed using the protocol of Ikeda et al. [23]. Briefly, the MAGIC-5 cells (5 × 10^5^ cells) were infected with the HIV_JRFL_ strain (100 ng of HIV p24) in the presence of 10 µM Tat-p2 peptide or 10 µM Tat-scrambled peptide and incubated for 24 h at 37 °C. Next, the cells were washed with PBS(−) twice and treated with DNase I (100 U; Takara) for 1 h. After DNase I treatment, the cells were washed with PBS(−) twice and incubated for 5 min at 37 °C in PBS(−) containing 0.25 % trypsin. After trypsinization, the cells were washed with PBS(−) twice and then digested in 200 μl of digestion buffer [10 mM Tris–HCl (pH 8.0), 150 mM NaCl, 10 mM EDTA, 0.1 % SDS, and 100 μg/ml proteinase K] for 2 h at 50 °C. After heat-inactivating proteinase K for 10 min at 95 °C, the lysate was subjected to phenol–chloroform extraction and ethanol precipitation. The resulting DNA pellet was resuspended in the distilled deionized water. To measure the amounts of late reverse transcription products, 2-LTR products and integrated (Alu) forms of viral cDNAs, the sample was examined by quantitative real-time PCR analysis using a primer pair specific for the R/gag, 2-LTR and Alu/gag region (M667, 5′-GGCTAACTAGGGAACCCACTG-3′; M661, 5′-CCTGCGTCGAGAGAGCT CCTCTGG-3′; 2-LTR sense, 5′-GAG ATC CCT CAG ACC CTT TTA G-3′; 2-LTR antisense, 5′-GTC AGT CGA TAT CTG ATC CCT G-3′; Alu-specific primer, 5′-TCCCAGCTACTCGGGAGGCTGAGG-3′; AA55, 5′-CTG CTA GAG ATT TTC CAC ACT GAC-3′). To further determine whether the amounts of late reverse transcription products are increased by the intracellular expression of the p2 peptide, MAGIC-5 cells (2 × 10^5^ cells) were transfected with pcDNA4/EGFP-p2x1. At 24 h post-transfection, EGFP-transduced and EGFP-p2x1-transduced MAGIC-5 cells were infected with the DNase I-treated single-round replication-defective virus (VSV-G-pseudotyped HIV_NL-CHΔenv WT_, 10 ng of HIV p24). At 24 h postinfection, the amounts of late reverse transcription products were measured as described above.

To investigate the effect of p2(Q6A) mutation on reverse transcription, MT-4 cells (2 × 10^5^ cells) or MDMs (2 × 10^5^ cells) were infected with the DNase I-treated virus (HIV_NL-CH p2(Q6A)_, 100 ng of HIV p24; VSV-G-pseudotyped HIV_NL-CHΔenv p2(Q6A)_, 10 ng of HIV p24). At 24 h post infection, the amounts of late reverse transcription products were measured as described above and expressed as a percentage (%) relative to the amounts in the control experiment.

### In vitro RT activity assay

To determine whether the p2 peptide could allosterically increase RT activity, the Tat-p2 peptide (GRKKRRQRRRARVLAEAMSQVTNTATIM), Tat-peptide (GRKKRRQRRRARVL), and p2 peptide (AEAMSQVTNTATIM) and a reverse transcription assay kit (F. Hoffmann-La Roche Ltd.) were used in this assay. Briefly, a solution containing 46 mM Tris–HCl, 266 mM potassium chloride, 27.5 mM magnesium chloride, 9.2 mM DTT, digoxigenin (DIG)-labeled dUTP, biotin-labeled dUTP, dTTP, and poly(A) × oligo(dT)_15_ template/primer hybrid was added to the reaction tube containing the HIV-1 RT standard (8.62 fmol) preincubated for 0.5 h with the Tat-p2 peptide, Tat-peptide, or p2 peptide at a ratio of 1:40, and then incubated for 1 h at 37 °C. After terminating the RT reaction, the reaction mixture was transferred to streptavidin-coated microtitre plates. DIG-labeled DNA was detected with an anti-DIG-POD conjugate, reacted with 2, 2-azino-di(3-ethylbenzthiazoline) sulfonic acid, and quantified by measuring OD at 405/490 nm. HIV-1-RT activity assay was performed as described in the kit protocol.

### Yeast two-hybrid plasmid construction and yeast two-hybrid library screening

The yeast expression plasmid pGBKT7 (bait) containing *TRP1* selection markers was used (Clontech). The HIV-1 p2 coding sequence from pNL4-3 was subcloned in-frame with the DNA-binding domain of the transcription factor Gal4 (Gal4 DNA-BD) into the *Eco*RI/*Bam*H1 sites of pGBKT7 to generate the pGBKT7-p2 plasmid. Before screening, the bait protein (GAL4 DBD/p2 fusion protein) was tested for its autoactivity in the absence of a prey HeLa S3 library. Human cDNA library screening was performed by mating the Y2Hgold expressing the bait protein with the Y187 strain pretransformed with the HeLa S3 (normalized) library encoded by the pGADT7 AD vector (Clontech) in accordance with the manufacturer’s protocol using double-drop-out (DDO) (without tryptophan and leucine with or without aureobasidin A and X-α-Gal) and quadruple-drop-out (QDO) (without tryptophan, leucine, adenine, and histidine and with or without aureobasidin A and X-α-Gal) selective medium agar plates. For cotransformation assay using the positive clones from the initial Y2H screening, the bait plasmid (pGBKT7-p2) and prey plasmid derived from the HeLa S3 cDNA library (pGADT7-MT-CO1) were cotransformed into Y2Hgold to validate the interactions in yeast. Furthermore, the alanine scanning mutagenesis in the highly conserved AEAMSQ motif of the p2 peptide in pGBKT7-p2 was used to determine the contribution of a specific residue to the interaction between the p2 peptide and MT-COI. The pGBKT7-p2, pGBKT7-p2(E2A), pGBKT7-p2(M4A), pGBKT7-p2(S5A), pGBKT7-p2(Q6A), and pGBKT7 vectors were cotransformed with pGADT7-MT-CO1 and plated on DDO/X and QDO/X/A. On the other hand, the bait plasmid (pGBKT7-53 or pGBKT7-Lam) was cotransformed into Y2HGold with the prey plasmid (pGADT7-T) to serve as a positive or negative control, respectively (data not shown). The AD/library cDNA inserts were sequenced and analyzed using the NCBI BLASTP program.

### Western immunoblot analysis

To determine the effect of the p2 peptide on MT-CO1, MAGIC-5 cells (3 × 10^5^ cells) were transfected with pcDNA4/EGFP-p2x1. At 48 h posttransfection, EGFP-transduced MAGIC-5 cells were lysed with 62.5 mM Tris–HCl, 2 % SDS, 10 % glycerol (pH 6.8), and 10 % 2-mercaptoethanol. The lysate was subjected to SDS-PAGE, and separated products were transferred to a polyvinylidene difluoride membrane. The membrane was stained with an anti-Xpress monoclonal antibody (Life Technologies, Inc.), an anti-MT-CO1 polyclonal antibody (Abcam), or an anti-actin monoclonal antibody (Oncogene™ Research Products). To monitor the processing of Pr55^*gag*^ into a mature capsid protein, the viral lysates from HIV_NL-CH p2(Q6A)_ were examined by western immunoblot analysis using an HIV-1-positive plasma.

### In vitro mitochondrial cytochrome c oxidase activity assay

To investigate the effect of the p2 peptide on the MT-CO positive control (Kit Item, KC310100-6) or native MT-CO in mitochondrial fractions from MAGIC-5 cells, we used an in vitro cytochrome c oxidase activity assay kit (BioChain Institute, Inc.) to measure the decrease in absorbance at 550 nm of ferrocytochrome c caused by its oxidation to ferricytochrome c by MT-CO in the presence or absence of the p2 peptide (final concentrations = 0, 80, and 160 µM, AEAMSQVTNTATIM). To prepare the native MT-CO, mitochondria were isolated from MAGIC-5 cells (1 × 10^8^ cells) using a mitochondria isolation kit for cultured cells (BioChain Institute, Inc.) and treated with n-dodecyl β-D-maltoside, which is one of the few detergents that maintain the cytochrome c oxidase dimer in solution at a low detergent concentration, thereby maintaining enzyme activity. Enzyme activity was determined colorimetrically by monitoring the oxidation of reduced cytochrome c as an absorbance decrease at 550 nm using a UV–VIS recording spectrophotometer (SHIMAZU). To further investigate the direct effect of the p2 peptide on mitochondria-derived MT-CO, sodium azide was used because it is commonly used in vitro as a rapid and reversible inhibitor of MT-CO [24, 25]. The effect of the p2 peptide (160 µM) on mitochondria-derived MT-CO pretreated with 10 mM sodium azide for 15 min was also examined in the same way.

### Confocal microscopy

HeLa cells (5 × 10^4^) were seeded in 400 μl of medium/well on 8-well chambered Lab-Tek II chamber slides (Thermo Fisher Scientific Inc.). After 24 h, the cells were transfected with pEGFP-p2x1, pEGFP-p2x9, or pEGFP-p2x17 using Lipofectamine LTX (Life Technologies, Inc.). After 24 h posttransfection, the transiently transfected MAGIC-5 cells were incubated for 0.5 h in a medium containing 200 nM MitoTracker (Life Technologies, Inc.) and washed with PBS(−). The cells were fixed by incubation with 200 μl of 1 % paraformaldehyde for 15 min, washed with PBS(−), and incubated in a cold 100 % methanol for 10 min. After that, the cells were stained with 4′, 6-diamidino-2-phenylindole (DAPI) for 5 min. The slides were mounted in VECTASHIELD (Vector Labs). Images were taken using a ConfoCor3 ZEN2009 confocal microscope (Carl Zeiss, Inc.).

### Proteinase K digestion of isolated mitochondria

MAGIC-5 cells were transfected with pEGFP-p2x1 using Lipofectamine LTX (Life Technologies, Inc.). After 24 h posttransfection, the mitochondria were freshly isolated using a mitochondria isolation kit (BioChain Institute, Inc.). The mitochondria were incubated in 20 µl of storage buffer included in the kit (BioChain Institute, Inc.) alone or in the presence of proteinase K (0.05 μg/ml). After 30 min of incubation, 20 μl of SDS-loading buffer was added and the mixture was then boiled for 5 min and examined immediately by Western immunoblot analysis using an anti-Xpress antibody (Life Technologies, Inc.), anti-Bcl-2 antibody (Santa Cruz Biotechnology, Inc.), or rabbit anti-MT-COI polyclonal antibody (Abcam Plc.).

### Determination of cytosolic ATP

The ATP Bioluminescence Assay kit CLS II (Roche Diagnostics Corporation) was used in accordance with the manufacturer’s recommendations to analyze the effect of HIV infection on intracellular ATP content. MT-4 cells (1 × 10^5^ cells) were infected with HIV_NL-CH_ or HIV_NL-CH p2(Q6A)_ (100 ng of p24 antigen) and cultured for 12 h at 37 °C. The cells were washed with PBS(−) twice and then digested with the cell lysis reagent included in the kit. ATP content was measured using the ATP Bioluminescence Assay kit CLS II in accordance with the manufacturer’s instructions. To further examine the effect of the p2 peptide on intracellular ATP level in MDMs, the cells (4 × 10^5^ cells) were infected with HIV_AD8_ or HIV_AD8 p2(Q6A)_ (500 ng of p24 antigen). Intracellular ATP level was measured in the same way.

## Abbreviations

HIV-1: human immunodeficiency virus type 1
CA: capsid
NC: nucleocapsid
MT-CO: mitochondrial cytochrome c oxidase
MT-CO1: MT-CO subunit I
MDMs: monocyte-derived macrophages
MA: matrix
Y2H: yeast two-hybrid
PIC: preintegration complex
FCS: fetal calf serum
DDO: double-drop-out
QDO: quadruple-drop-out
